# On how to incorporate public sources of situational context in descriptive and predictive models of traffic data

**DOI:** 10.1186/s12544-021-00519-w

**Published:** 2021-11-25

**Authors:** Sofia Cerqueira, Elisabete Arsenio, Rui Henriques

**Affiliations:** 1 LNEC I.P. & INESC-ID, Instituto Superior Técnico, University of Lisbon, Lisbon, Portugal; 2 Department of Transport, LNEC I.P., Lisbon, Portugal; 3 INESC-ID, Instituto Superior Técnico, University of Lisbon, Lisbon, Portugal

**Keywords:** Sustainable mobility, Data science, Big data, Public transport, Situational context, Multimodality

## Abstract

**Background:**

European cities are placing a larger emphasis on urban data consolidation and analysis for optimizing public transport in response to changing urban mobility dynamics. Despite the existing efforts, traffic data analysis often disregards vital situational context, including large-scale events, weather factors, traffic generation poles, social distancing norms, or traffic interdictions. Some of these sources of context data are still private, dispersed, or unavailable for the purpose of planning or managing urban mobility. Addressing the above observation, the Lisbon city Council has already established efforts for gathering historic and prospective sources of situational context in standardized semi-structured repositories, triggering new opportunities for context-aware traffic data analysis.

**Research questions:**

The work presented in this paper aims at tackling the following main research question: How to incorporate historical and prospective sources of situational context into descriptive and predictive models of urban traffic data?

**Methodology:**

We propose a methodology anchored in data science methods to integrate situational context in the descriptive and predictive models of traffic data, with a focus on the three following major spatiotemporal traffic data structures: i) georeferenced time series data; ii) origin-destination tensor data; iii) raw traffic event data. Second, we introduce additional principles for the online consolidation and labelling of heterogeneous sources of situational context from public repositories. Third, we quantify the impact produced by situational context aspects on public passenger transport data gathered from smart card validations along the bus (CARRIS), subway (METRO) and bike sharing (GIRA) modes in the city of Lisbon.

**Results:**

The gathered results stress the importance of incorporating historical and prospective context data for a guided description and prediction of urban mobility dynamics, irrespective of the underlying data representation.

Overall, the research offers the following major contributions:

1. A novel methodology on how to acquire, consolidate and incorporate different sources of context for the context-enriched analysis of traffic data;
2. The instantiation of the proposed methodology in the city of Lisbon, discussing the role of recent initiatives for the ongoing monitoring of relevant context data sources within semi-structured repositories, and further showing how these initiatives can be extended for the context-sensitive modelling of traffic data for descriptive and predictive ends;
3. A roadmap of practical illustrations quantifying impact of different context factors (including weather, traffic interdictions and public events) on different transportation modes using different spatiotemporal traffic data structures; and
4. A review of state-of-the-art contributions on context-enriched traffic data analysis.

The contributions reported in this work are anchored in the empirical observations gathered along the first stage of the ILU project (see footnote 1), providing a study case of interest to be followed by other European cities.

## Introduction

European and worldwide cities are entailing complex urban mobility changes to respond to the COVID-19 pandemic crisis and satisfy climate and environmental goals. Urban mobility decarbonisation initiatives, encompassing enhanced public transport, infrastructure interventions towards active modes such as walking and cycling, are aimed to provide a better fit with individual mobility needs and the distribution of traffic generation poles. In this light, most European cities such as Lisbon in Portugal are pursuing technological solutions to face the challenge of dynamically adapting public passenger transportation systems in accordance with the real traffic dynamics. To this end, efforts are being established towards traffic data collection, consolidation and exploration from public and private, individual and active modes of transport.

Traffic dynamics are situated, meaning that these are dependent on a high multiplicity of situational factors including spatial urban context (particularly traffic generation and attraction poles, including commercial, business, education, or leisure areas), meteorological context, occurring events (including sport matches, concerts or congresses), calendrical context, or traffic interdictions (including road accidents and construction works). Despite their well-recognized impact on urban mobility, sources of urban context are commonly disregarded for two major reasons. First, some of these sources are unstructured, private or unavailable, preventing their automated consolidation. Second, existing principles for context-aware traffic data analysis remain largely dispersed. In fact, state-of-the-art contributions for context-aware descriptive and predictive tasks generally fail to model the joint impact that these multiple sources of context exert on urban mobility. In addition, existing works generally fail to separate the important role of both historical and prospective sources of context. For instance, historical weather and event data are important to assert the true impact of situational context in transport demand, while prospective events and weather forecasts are essential to support predictions.

The research work presented in this paper aims at tackling the research question: How to incorporate historical and prospective sources of situational context into descriptive and predictive models of urban traffic data? To this end, we propose a comprehensive set of principles for the dynamic acquisition and incorporation of situational context onto traffic data analytics. We tackle the problem of context-augmented traffic data analysis in accordance with three major research needs:

1. how to perform context-aware analysis for different spatiotemporal traffic data structures, including georeferenced time series data, origin–destination (OD) tensor data, and raw event data (e.g. smart card validations on the public transport network);
2. how to dynamically consolidate the different sources of situational context; and
3. how to adapt the learning in accordance with the targeted task, whether descriptive modelling, pattern discovery, anomaly detection or forecasting.

The Lisbon city is used as a case study for assessing the proposed research contributions. In recent years, the Lisbon City Council (CML) established protocols to monitor multiple sources of traffic data and situational context. Heterogeneous sources of situational context data are being consolidated in semi-structured repositories, offering unique opportunities for context-aware traffic data analysis. The contributions reported in this work are anchored in the empirical observations gathered along the first stage of the ILU project^1^, providing a study case of interest to be followed by other European cities.

The gathered results consider bus, subway and cycling modes of urban traffic in the Lisbon city to quantify the impact produced by context data sources, showing the corresponding correction factors and motivating their relevance for pursuing context-aware analysis to support urban mobility planning decisions.

Accordingly, this work offers the following major contributions:

1. A novel methodology on how to acquire, consolidate and incorporate different sources of context for the context-enriched analysis of traffic data;
2. The instantiation of the proposed methodology in the city of Lisbon, discussing the role of recent initiatives for the ongoing monitoring of relevant context data sources within semi-structured repositories, and further showing how these initiatives can be extended for the context-sensitive modelling of traffic data for descriptive and predictive ends;
3. A roadmap of practical illustrations quantifying impact of different context factors (including weather, traffic interdictions and public events) on different transportation modes using different spatiotemporal traffic data structures; and
4. A review of state-of-the-art contributions on context-enriched traffic data analysis.

This paper is structured as follows: Sect. 2 introduces essential background; Sect. 3 surveys related work on context-enriched data analysis; Sect. 4 combines existing and novel principles within a coherent methodology for context-aware traffic data analysis; Sect. 5 presents main results from using the Lisbon mobility data highlighting the relevance of incorporating sources of situational context; Sect. 6 concludes through presenting final remarks.

## Background

This section provides a structured view on traffic data analysis (Sect. 2.1), identifies major sources of situational context data (Sect. 2.2), and introduces the Lisbon’s urban ecosystem as our case study (Sect. 2.3).

### Traffic data analysis

Four major sources of traffic data are generally monitored in urban centres: (1) road traffic data produced by stationary devices positioned along the city, and geolocalized speed meters from active mobile devices); (2) automated fare collection (AFC) data from public transport operators, generally consisting of smart card validations from users at stations or vehicles; (3) public transport planning data, generally termed General Transit Feed Specification (GTFS) data, encompassing all information pertaining to schedules, routes, stations of the public carriers in a standardized format; and (4) individual trajectory data collected from mobile devices during active modes of transport.

**Fig. 1:**
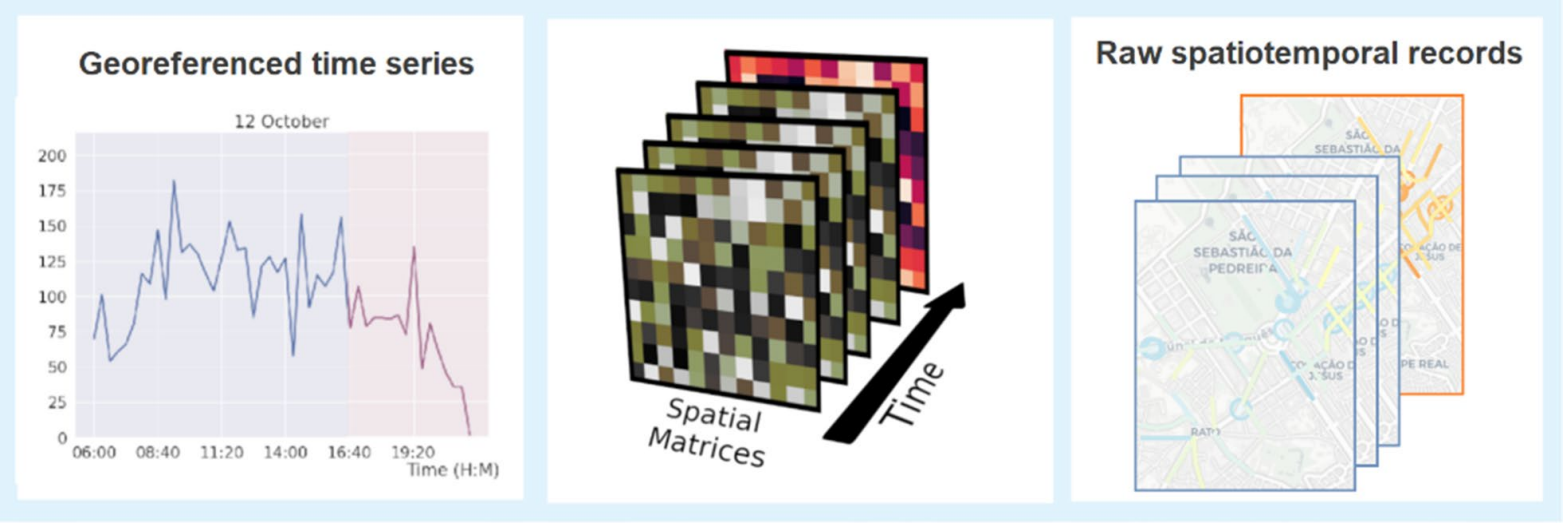
Targeted spatiotemporal traffic data structures: georeferenced time series, origin–destination tensors, and raw events (individual/aggregated trajectories and card validations)

The above traffic data structures are generally mapped into new spatiotemporal data structures more conducive to the subsequent data analysis. Generally, we find three major representations of traffic data (Fig. 1):

- Georeferenced time series data (Sect. 2.1.1), generally providing heterogeneous views on traffic dynamics along fixed intervals of time, such as passenger volume (card validations) at a given station or road traffic speed and frequency at a particular road junction;
- Origin–destination tensor data (Sect. 2.1.2) mapped from paired entry-and-exit card validations of users along the public transport network, as well as from trajectories produced from road and active modes of transport;
- Raw event data (Sect. 2.1.3), comprising three major types of mobility events: (1) smart card validations, (2) events associated with road traffic congestions, and (3) individual trajectories.

#### Traffic time series analysis

Traffic data can generally be represented as georeferenced time series data, pairs (time series, coordinates).

Given a time series${\mathbf{x}}_{1.. T}$, descriptive tasks aim at extracting patterns or modelling${\mathbf{x}}_{1.. T}$, while predictive tasks generally aim to forecast the *h* upcoming observations,${\mathbf{x}}_{\mathrm{T}+1 ..\mathrm{ T}+\mathrm{h}}$, from available observations${\mathbf{x}}_{1.. T}$,. These tasks typically focus on a single variable at a time, being complementary variables in multivariate time series used to aid the description or prediction of the target variables.

Classical approaches for time series analysis generally rely on statistical principles, including decomposition, auto-regression, differencing, exponential smoothing, and smooth transitioning functions [7, 45] (Diijk 2002). Machine learning approaches for time series analysis generally segment the series to compose a dataset to guide the learning of the descriptive and predictive models under a specific loss criterion. Distance-based approaches and recurrent neural network learning are paradigmatic examples [3, 4, 16].

#### Origin–destination traffic data analysis

Smart card validations or individual trajectory data can be mapped into origin–destination (OD) tensors, generally a numeric three-dimensional ${location}_{origin} \times {location}_{destination} \times variable$ matrix inferred along a specific region and time period [26]. Origin and trip destinations are generally given by some spatial criterion of interest (e.g. stations, zoning scheme, or geographical mesh). Variables generally reveal information pertaining to origin–destination trips, typically the total volume of individuals or vehicles moving along each OD pair.

In the context of automated fare collection data, entry and exit validations per transport operator are generally paired to identify a trip segment. As a single trip may require commutes within a single carrier or between different carriers, trip segments can be then properly concatenated under specific spatiotemporal assumptions to identify the true origin and destination of a single whole trip [48]. Similar principles are commonly applied to individual trajectory data, where the time spent by individuals at a single location or untracked periods should provide the desirable splitting-concatenating criteria. OD tensors can be either inferred from different calendar periods (such as weekdays or holidays), possibly spanning an arbitrarily-high number of days. This possibility gives rise to two different types of OD tensor data structures [44]: (1 single OD tensor whose variables (e.g. passenger volume generally correspond to the daily average (e.g. daily passenger volume along the targeted time period, or (2 OD tensor series, an ordered set of OD tensors where each tensor generally corresponds to a single day or period within a day.

Given a single OD tensor, descriptive tasks generally aim at finding frequent patterns or detecting vulnerabilities [26]. For this latter task, and in the context of public transport, in addition to passenger volume, alternative statistics can be computed for each origin–destination pair, including mean and variance estimators of the (1) number of commuting trips per passenger, (2) overall travel time per passenger, (3) travel time for commuting trips per passenger, or (4) walking distance spent in commuting trips per passenger. In the context of predictive tasks, single OD tensors are generally assumed as ground truths for OD movements to be observed in the near future. To surpass the inability to capture trends and seasonal factors within single OD tensors, series of OD tensors can be alternatively considered for both descriptive and predictive ends [44]. In this context, an OD tensor series can be generally seen as a multivariate time series with a multivariate order as high as the number of origins, destinations or origin–destination pairs. In this context, the listed principles in Sect. 2.1.1 can be considered for extracting patterns, modelling fluxes and forecasting upcoming movements [16, 11, 45].

#### Raw traffic data analysis

An event is a tuple$e=(x, s, \tau )$, where $\mathbf{x}=({x}_{1},..,{x}_{m})$ is the observation, either univariate ($m=1$) or multivariate ($m>1$) depending on the number of monitored variables; $s$ is the spatial extent of the observation$\mathbf{x}$. The spatial extent s can be any spatial representation associated with the event, such as a geographic coordinate or a trajectory; and $\tau$ is the temporal extent of the observation$\mathbf{x}$, either given by a time instant or a time interval. A spatiotemporal event dataset is a collection of events, $E=\{{e}_{1}, {e}_{2}, .., {e}_{n}\}$, each event producing a (multivariate) observation recorded along specific spatial and temporal contexts. Notable examples of event traffic data are raw smart card validations where the observation identifies the user, the spatial extent is a coordinate (typically bound to a station) and temporal extent is the timestamp of the validation. Trajectory data can also be seen as event data, in this context the spatial extend corresponds to the trajectory, i.e. an ordered set of coordinates, and the temporal extent to the time interval where the trajectory was travelled.

Descriptive and predictive tasks from event data generally resort to one of the previously covered spatiotemporal data structures (Sects. 2.1.1 and 2.1.2). First, a spatial and temporal granularity can be fixed and estimators, such as counts and other statistics, applied to produce time series or OD tensors. Once event data is mapped into time series, the traditional modelling, motif analysis, novelty detection and forecasting approaches can be readily applied [23]. Additional approaches to learn descriptors and predictors from raw event data are nevertheless available, including distance-based and generative approaches from event-sets [14], dedicated neural processing architectures [6] or episode mining [47].

### Sources of situational context

The available sources of situational context consolidated by the Lisbon City Council and considered in the targeted traffic data analysis tasks include:

- Public events, including: (a) conventions, (b) festivals, (c) concerts, and (d) sport events. The historic and prospective events are currently sourced from two major sites: (1) the planned usage of large halls, stadiums and large open areas in the city of Lisbon, and (2) the cultural agenda of the city;
- Historic, current and 10-day forecasted weather record data sourced from three meteorological stations maintained by Instituto Português do Mar e da Atmosfera (IPMA). Weather variables with potential impact on traffic include temperature, humidity, wind intensity, precipitation, nebulosity, and visibility;
- Relevant occurrences in the city, including: (a) road accidents, (b) medical emergencies, (c) fires and floods, (d) logistical help and falling structures, (e) transport requests, (f) conservation and complaints, and (g) rescue and civil protection;
- Ongoing and planned construction road works (traffic conditioning events) characterized by a set of trajectories with (possibly non-convex) interval of obstruction and accompanying details (including the number of affected ways and whether interruption is spasmodic);
- Urban planning of the city with the localization of traffic generation-attraction poles, including: (1) [*commercial*] malls, commercial permits, markets, terminals; (2) [*utils*] citizen spaces, conservatories, social stores, councils, parks, public authorities; (3) [*education*] public and private schools, universities, institutes; (4) [*health*] hospitals, health centres, clinics; (5) [*sport*] sport facilities; (6) [*cultural*] concert halls, monuments, museums, movie; theatres, amphitheatres; (7) [*transportation*] public transport networks; and (8) [*leisure*] recreational spaces;
- Other sources of interest including context around active modes of mobility (such as cycling roads and maps with the walking potential of urban roads and street segments), as well as calendrical context encompassing annotations associated with academic breaks, festivities, holidays and weekly-monthly-yearly seasonal factors.

### Case study: Lisbon city

The Lisbon city Council has established numerous initiatives to pursue a sustainable urban mobility. In the context of urban data monitoring and consolidation, the following sources of traffic data are currently being consolidated:

- Automated fare collection data from the public transportation network, including card validations and the GPS positioning of public vehicles;
- Bike sharing data from the Lisbon’s public bike sharing system (GIRA^2^), including trip records per user, user feedback on bicycle’s condition, bike charging information, bike malfunction and repair status, among others;
- Road traffic data from three major types of sensors: (1) inductive loop detectors in major road junctions in the city, offering discrete views on traffic flow; (2) individual mobile devices with GPS producing aggregated views on traffic congestions (geolocalized speed data), explored in the context of partnerships with WAZE and TomTom; and (3) privacy-compliant cameras in main roads;
- Other sources: data pertaining to emerging modes of transportation, including private scooter traffic data. An entry requirement for new private operators is precisely the full disclosure of trip records.

The public transport network in the Lisbon Metropolitan Area (LMA) which includes the Lisbon city council and more 17 municipalities, covers more than 12 transport operators, being CARRIS (the major bus operator in the City of Lisbon) and METRO (the subway operator) distinctively large in passenger volumes and the only modes offering a solid footprint coverage within the Lisbon municipality. The single public bike sharing system is operated by EMEL/GIRA. Apart from active modes of transportation, the providers of bus, subway, railway and inland waterway modes of transport are currently operating under an integrated fare collection system, enabled through the VIVA card initiative. The VIVA card initiative, firstly established between METRO and CARRIS, currently encompasses other carriers interfacing with the city of Lisbon for an operation under a single consolidated ticketing system.

## Related work

Recent attention has been paid to the incorporation of contextual data to enhance the understanding of mobility dynamics and support traffic data analysis [19]. Usually these factors are divided on whether they can be planned [18]—including football matches, concerts, festivals, construction works, urban planning—or not [36]—weather, air quality, traffic accidents, emergencies. The former factors are often mentioned as Planned Special Events (PSE), as earlier introduced by Latoski et al. [18]. Some of the challenges of integrating spatiotemporal contextual information and its role in the development of smart cities are discussed by Sagl et al. [34]. Different types of situational context have been considered in previous research works aiming at analysing traffic dynamics, namely: weather and occurrences of potential relevance from Twitter data [36],accident and weather records, sport matches, festivals, and other crowded events inferred from social media data recurring to natural language processing techniques [17, 33, 46]. Tempelmeier et al. [38] introduced an approach for enriching traffic data with available sources of situational context exclusively retrieved from the web, including events from social media, weather portals, and traffic warnings.

Two major classes of context-sensitive approaches for traffic data analysis can be identified from the existing literature. First, approaches that aim to describe and predict traffic dynamics by segmenting data into chunks according to the available situational context and using only context-specific chunks for understanding and forecasting demand. Second, approaches able to embed the context directly into the models by capturing correlations with the context and using these correlations as corrections to automatically adjust descriptive and predictive models.

### Context-selective analysis

Context-selective analysis of traffic data segments the available data—whether time series, OD series or raw traffic events—into chunks, each chunk sharing similar context. Data chunks are then used with the purpose of learning context-sensitive descriptive and predictive models. Hence, we can see descriptors and predictors of traffic as a set of arbitrarily different context-specific models. More advanced structures can, however, be considered [20]. For instance, Li et al. [21] proposes the use of decision trees where the branches are placed in accordance with the calendrical contextual data and the roots contain context-specific models.

Kwoczek et al. [17] proposed a method to predict and visualize traffic congestion caused by planned special events. Public events are generally characterized by two waves of congestion: people arriving and leaving the event. The authors recognized the difficulty of estimating the impact of these waves (the popularity of event) and, to address this observation, developed a distance-based approach to predict the waves using nearest neighbours from past PSEs, showing that event-sensitive predictions yield improvements. The presented solution relies heavily on historical event data which can be scarce.

El-Assi et al. [9] provide a multi-level model considering the impact of land use, built environment, and weather measures on bike share ridership. Similarly, Tran et al. [42] consider the problem of predicting bike sharing system flow. To this end, they propose the use of a regression model and further consider the effects from five categories of context variables: public transport, socio-economic, topographic, bike-sharing network, and leisure variables.

### Context-based corrections

The second option is to allow the learning to analyse context and its integration with traffic data, thus allowing the available context to shape the learned models. Gallop et al. [10] explore complex serial correlation patterns between weather and bike traffic and use this effect to adjust the error terms of the classic autoregressive integrated moving average (ARIMA) models. They further suggest that this correction can be used to affect historical data in an effort to create context-independent models to facilitate specific traffic data analysis tasks.

Rodrigues et al. [33] introduced a Bayesian additive model (BAM) to predict the number of public transport trip arrivals in a given place. By employing a Bayesian additive framework, the proposed model is also capable of decomposing a time series into components that reflect the contributions of routine behaviour and individual special events. The authors formulate the problem as a model-based machine learning approach, which seeks to create a model tailored to this particular problem, instead of using standard algorithms and transform the problem to fit them. The model is built on the assumption that a base routine component and a variable number of event components exist. The components are summed to obtain the total number of observed arrivals in a given area. Variables used to parameterize the algorithm include the start/end time of trips, its duration, if it is a multi-day event or not and the event’s category. To extract the event’s category, they propose an approach that uses results from web search engines. Rodrigues et al. [33] show that this approach outperforms other models that integrate context in their predictions. The proposed method has the additional advantage of giving information of each individual event’s influence, making the model highly interpretable.

To analyse the impact of social events, Tomaras et al. [41] propose the use of a metric, influence factor, to measure the influence of an event in the nearby bike station. The influence factor is a ratio between sum the drop-off and pick-up when an event happens and drop-off and pick-up in a typical day. This factor can be used as a correction factor for descriptive and predictive models.

Ashqar et al. [1] consider a data-fusion approach towards the analysis of context-enriched bike demand. They propose the use of random forests to rank context predictors and consider them to develop a forecasting model using a guided forward step-wise regression approach. They found that time-of-day, temperature and humidity are significant predictors.

Thomas et al. [39] studied cycle flows from utilitarian and recreational paths in the Netherlands. A bi-level model for predicting the demand for cycling was used. The lower level describes how cyclists value the weather. The upper level is the relation between demand and this weather value. Most fluctuations are described by the model.

Principles to incorporate context within neural networks have been additionally proposed. Thu et al. [40] propose multi-layer perceptron regressors from multi-source context data to predict bike pick-up demands in New York city considering clusters of stations based on their geographical locations and transition patterns. The proposed networks combine weather factors (temperature, wind speed, and visibility) and taxi trip records. Despite its relevance, temporal dependencies between observations are disregarded. Pan et al. [29] incorporate weather record data at the input layer of LSTMs to improve the prediction of bike sharing demand for balancing of distribution of bikes across stations. Results evidenced improvements against context-unaware LSTMs. Recent contributions on deep learning research also show the possibility of incorporating specific forms of calendrical and spatial awareness [5, 30].

## Principles for context-aware analysis of traffic data

**Fig. 2:**
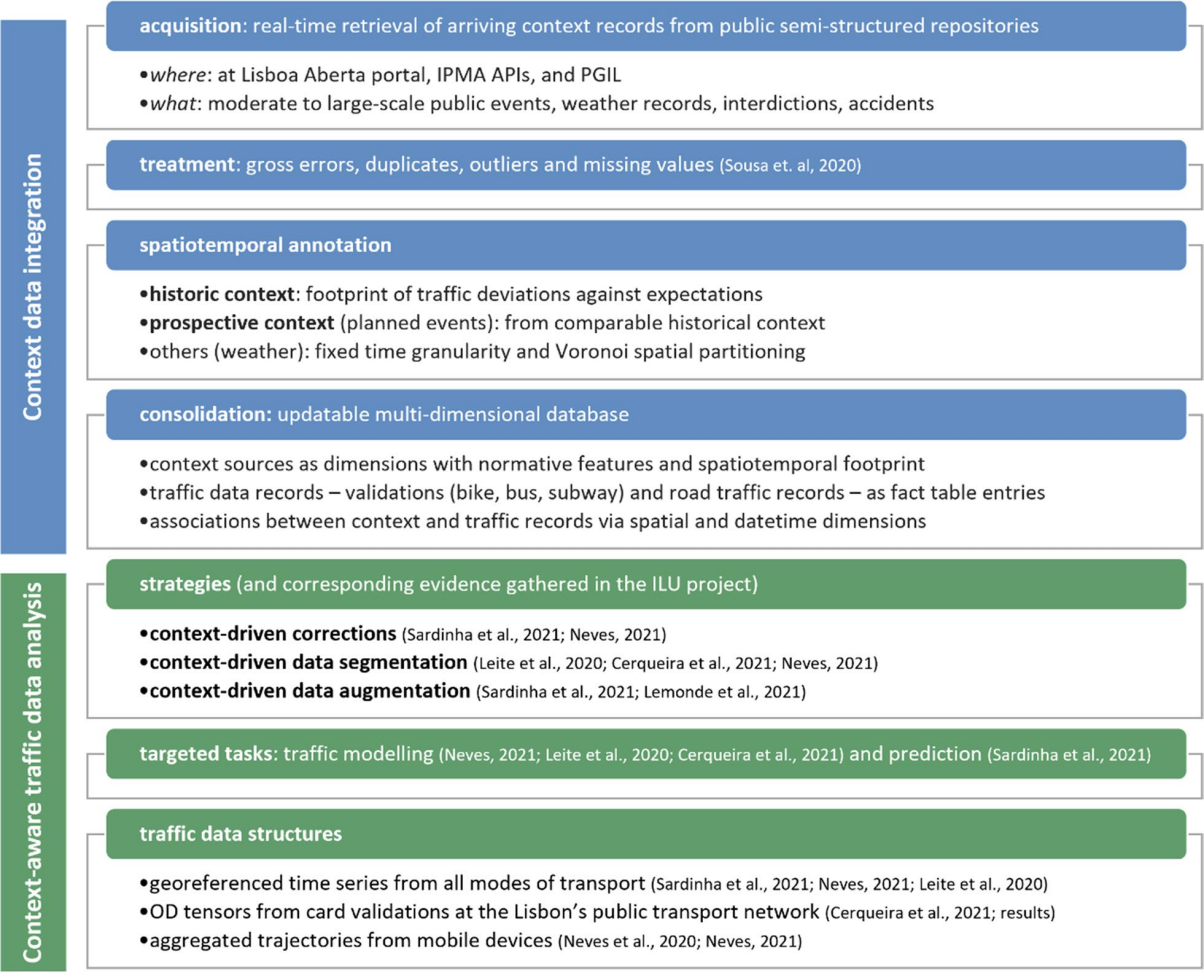
Pursued principles along the proposed two-step methodology for the context-aware analysis of multimodal traffic data in the Lisbon city

Anchored in the surveyed state-of-the-art contributions and practical experience from the established R&D efforts with the Lisbon city Council, this section introduces a methodology for the incorporation of context in big traffic data analytics (Fig. 2). The methodology is composed of two major steps: (1) acquisition, standard annotation and consolidation of heterogeneous sources of context (Sect. 4.1); followed by the (2) augmentation of the subsequent computational approaches for the descriptive and predictive modelling of traffic modes (Sect. 4.2).

### Automated acquisition and consolidation of context data

Two major principles are proposed for the automated acquisition of situational context. First, social media, public administration repositories, weather portals, online calendars of festivities, cultural agendas, theatre sites, and online news can be periodically explored with the aim of retrieving specific context sources of interest. Wibisono et al. [46], Tempelmeier et al. [38] and Tang et al. [] placed principles towards this end. Despite the importance of web data mining, the acquisition of situational context data from the web is generally subjected to uncertainties related with data quality and availability. In addition, information pertaining to relevant city occurrences (such as road interventions and other significant events) are generally dispersed across different sources and often unstructured, being hard to infer the spatial and temporal extent of influence from these complementary sources of context.

**Fig. 3:**
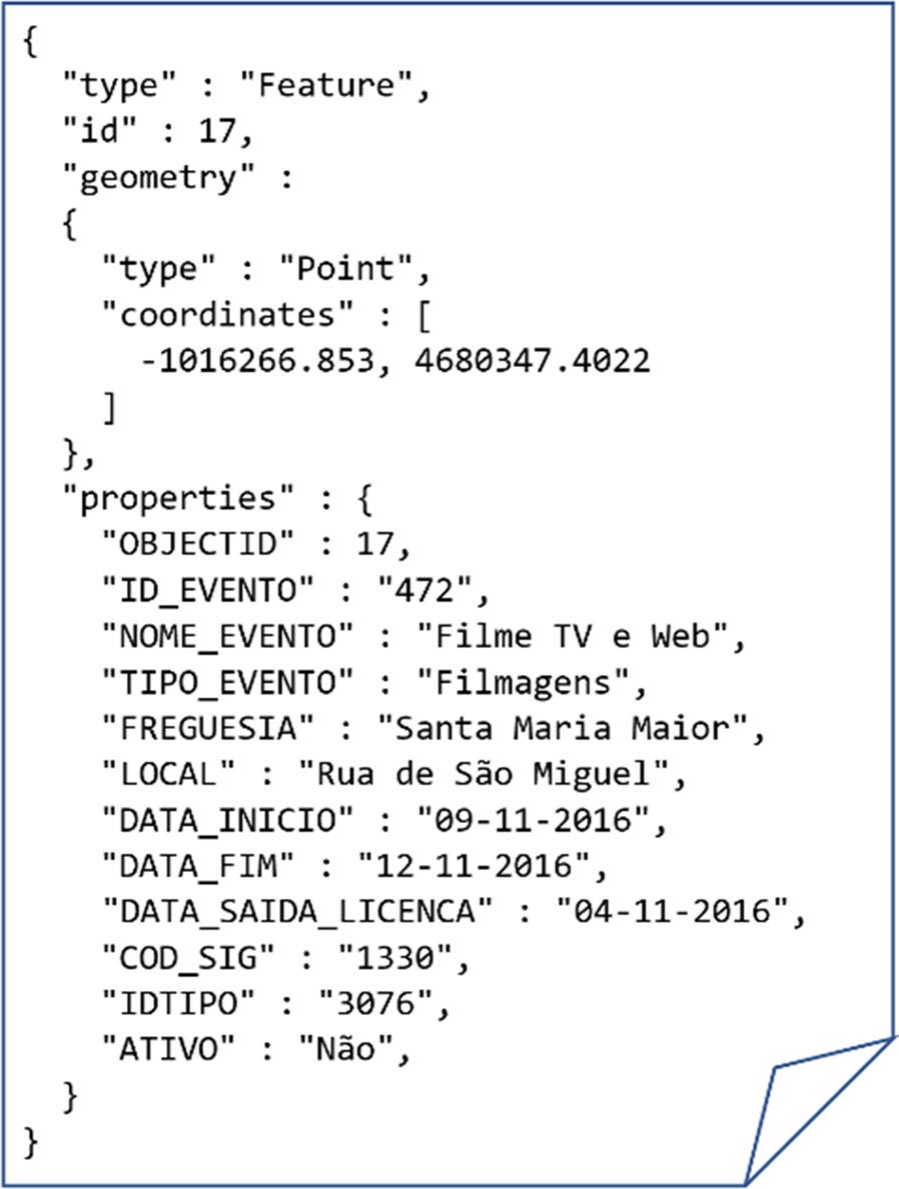
Illustrative interdiction with the road trajectory and time ranges of applicability specified in geoJSON format

Second, in cities with well-established efforts towards the gathering and provision of situational context, the listed challenges on the acquisition step can be addressed so that the sourced context information is less susceptible to the uncertainty factors related with unstructured content. In this context, periodic routines can be executed to extract context from structured or/and semi-structured sources maintained by the city Councils and other entities. Illustrating, the Lisbon city Council (CML) stores the sources of situational context listed in Sect. 2.2 using semi-structured data representations (JSON) at the “*Lisboa Aberta*” (Open Lisbon) portal. The repositories are periodically updated in order to facilitate administrative tasks, as well as to potentiate complementary strategic and research initiatives. The repositories can be standardly accessed, and the monitored contents are maintained in standard JSON formats (Fig. 3) in order to facilitate structured searches and the recording of geographical information, taking opportunity of geoJSON facilities for the normalized specification of locations, perimeters and trajectories. As a result, the dynamic retrieval and labelling of context data can be done in a fully automated fashion [19]. Some of these public sources of context may further entail preprocessing procedures to guarantee the absence of errors and the adequate imputation of missing entries. For instance, faulty weather records on specific points in time can be estimated using measurements from nearby stations or adjacent observations in time.

Sources related with planned events can be automatically annotated in accordance with their typology and duration. The spatial extent, as well as the historical and prospective duration of some of these events, such as construction works, is maintained in these repositories. In contrast, for public events without such information, rules can be dynamically inferred with expectations on the average event duration in accordance with its typology. An illustrative rule is that sport events approximately impact entry validations at public transport stations 120 min before the game and up to 60 min after the game. Context-specific deviations to traffic can be assessed against daily traffic expectations to determine the spatiotemporal extent of the event. The deviation of demand can be assessed by considering an envelope (statistical bounds) over a model of traffic demand in accordance with the principles highlighted in one of our previous works [19]. In the context of historical public events, the dynamic detection of the time interval and locations with deviating demand produces the necessary annotations for these traffic records to be carefully segmented or corrected in the context of traffic data analysis. Considering predictive traffic analytics, the annotation of prospective public events can be estimated by retrieving the spatiotemporal extent of comparable events—e.g. sport matches in the same stadium during similar hours or public concerts in the same hall on the same weekday. The annotation of events with the corresponding spatiotemporal extent is essential to signal subsequent traffic data analysis, preventing their impact to undesirably impact both descriptive and predictive models.

**Fig. 4:**
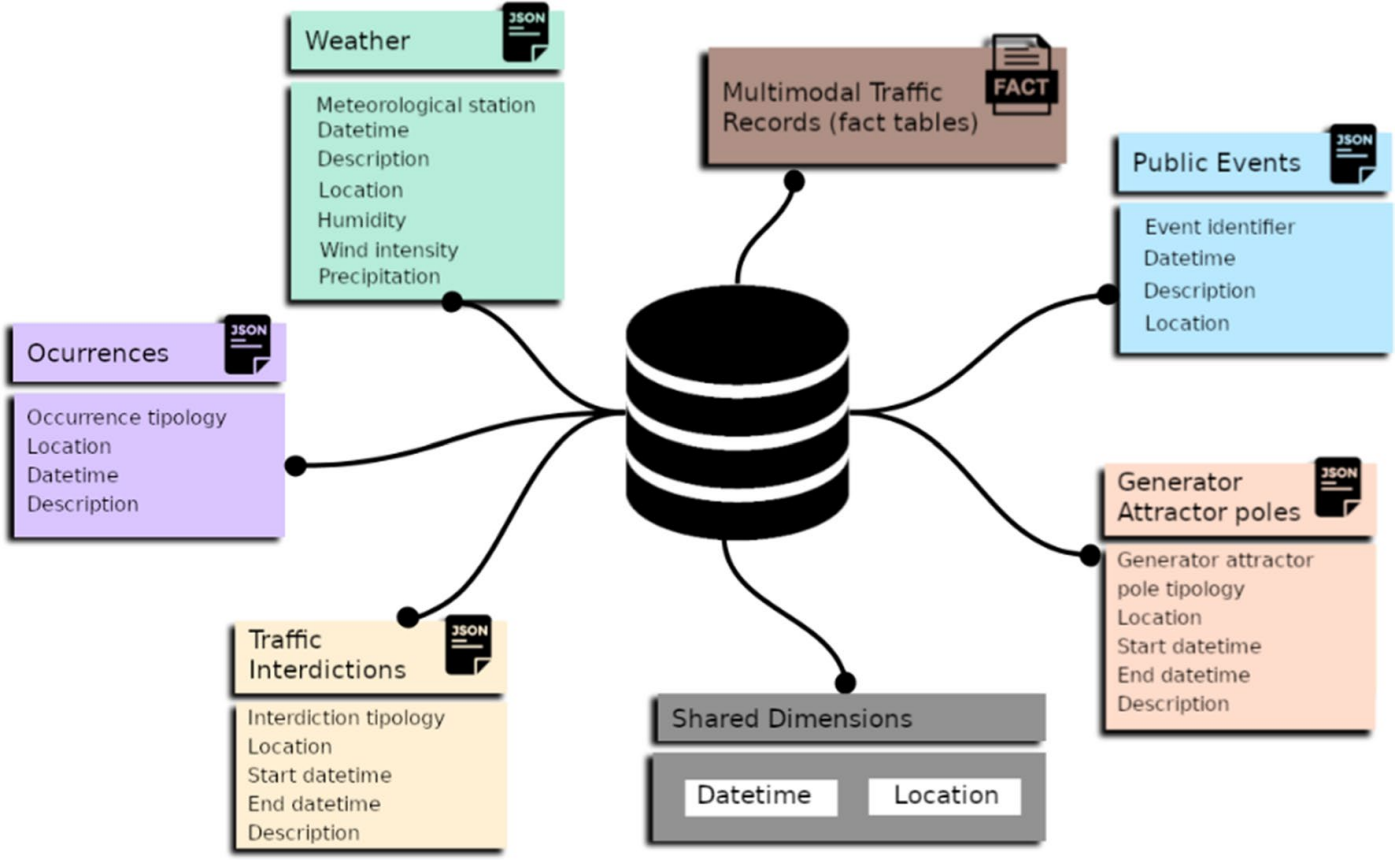
Multi-dimensional schema where each context data source is stored in a dedicated dimension, and traffic records are stored in fact tables. Associations between a fact entry and the corresponding entries for each context dimension are efficiently established by assessing the location (trajectory) and timestamp (time interval) of a traffic record against the spatiotemporal footprint of a context entry (e.g. public event, weather record, interdiction)

The available sources of context data and traffic data are consolidated in a multi-dimensional database [25]. Traffic records (whether associated with point, origin–destination or trajectory annotations) are stored in dedicated fact-tables and linked to shared dimensions, including time, space and mode-operator dimensions. Context data is further stored in dimension-tables and linked to fact entries (see Fig. 4). Illustrating, weather records are stored in a dedicated dimension and linked traffic records based on their measurement time and spatial validity. Similarly, planned events are stored in a dedicated dimension and linked to traffic records according to well-established spatiotemporal criteria. This consolidation step enables a coherent navigation throughout the records along different geographies, time periods and modes of transport. Data extraction facilities should be able to adequately index spatial, temporal and modal information for the efficient retrieval of both traffic and context data.

### Context-aware traffic data analysis

The integrative analysis of traffic data against the available context offers unique opportunities to understand and anticipate traffic dynamics. To this end, statistically significant correlations between mobility dynamics and their situational context can be first identified and then used to enhance the targeted descriptive and predictive models.

Under the previously suggested consolidation schema, comprehensive correlations can be found between traffic records and their accompanying situational context using traditional Pearson or Spearman indices, as well as principles from multi-dimensional subspace clustering and relational pattern mining [8, 13, 15]. Illustrating, as extreme weather conditions considerably affect public cycling demand, weather-demand correlation can be assessed for a context-guided modelling of traffic dynamics (see Sect. 5.4). Once correlations are identified, they can be used as correction factors to automatically adjust descriptive and predictive models [10, 41].

In alternative, traffic data can be segmented in accordance with the available situational context—traffic records under comparable events and calendrical, meteorological and spatial context. Context-specific slices can be then selected for learning context-specific descriptors and predictors [9, 21].

Finally, the available context can be seen as additional variables, whether static or temporal, used to augment traffic data. The context-enriched traffic data can then be subjected to machine learning approaches able to deal with heterogeneous, multivariate and spatiotemporal data structures. Neural network approaches are a natural candidate towards this end, as they can receive an arbitrarily-high number of inputs and be architecturally optimized to capture their inherent temporal, spatial and modal dependencies [5, 30, 35, 40].

The application of these three groups of principles—*context-driven corrections, context-driven data segmentation* and *context-driven data augmentation*—can be pursued irrespectively of the underlying data structure. Sections 4.2.1–4.2.3 instantiate some these principles based on empirical evidence gathered from the ILU project, and complement them with dedicated strategies for the context-aware analysis of time series, OD and traffic event data.

#### Context-aware analysis of traffic time series

Modelling and forecasting traffic demand are central tasks. In the surveyed work, different principles were recovered to support these tasks in the presence of situational context [1, 17, 33, 39, 42]. *Context-driven corrections* have been proposed for both classical and machine learning approaches for time series analysis. *Context-driven data segmentation* is not suggested for classical descriptive and predictive approaches, such as SARIMA and Holt-Winters, due to the observed temporal discontinuities with arbitrarily high duration produced by the segmentation procedures.

In the context of the ILU project, *context-enriched neural network approaches* were proposed by extending recurrent neural network layering to incorporate both historical and prospective sources of context [35]. One of the best performing neural architectures for the urban traffic data in Lisbon is a sequential composition of long short term memory (LSTM) components and/or gated recurrent units (GRU). To incorporate historical sources of context data, we take advantage of the fact that LSTMs are inherently prepared to learn from multivariate time series with an arbitrarily high order. In this way, context variables can be combined at the input layer to guide the learning task by relying on masking principles. The masking principle for context incorporation is illustrated in Fig. 4. For instance, calendric masks can be used to identify weekdays or academic periods and breaks; situational masks mark periods where events of interest may impact the demand observed at a given geography; and weather masks contain as many variables as weather attributes of interests.

**Fig. 5:**
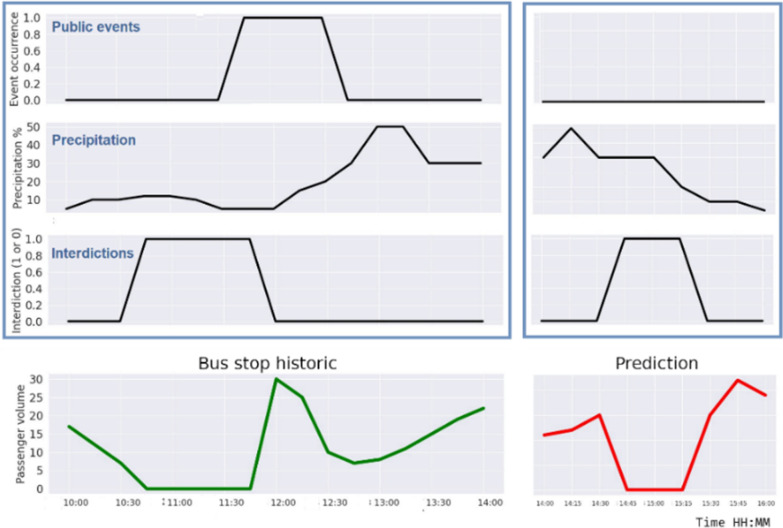
Context masks: increasing the multivariate order of the target traffic time series data with context variables, including calendrical, meteorological and spatial variables [35]

Prospective sources of context, including weather forecasts or planned events, may be available along the horizon of prediction. Considering the introduced sequential composition of LSTMs as the target architecture, prospective context data can then be inputted into the last LSTM component to adjust predictions (see Fig. 5). In this way, prospective context can be used as a denoiser or regularizer of the forecasted time series within predictive tasks.

#### Context-aware analysis of OD tensor data

Two major groups of principles are devised in accordance with the two major OD data structures introduced Sect. 2.1.2: (1) single OD tensor where trip statistics are inferred from a well-established time period, and (2) OD tensor series where the statistics pertaining to the monitored OD trips are inferred from multiple periods under a sliding time windows.

Context-aware tasks along the first scenario generally aim at assessing the impact of a well-defined context factor (such as an historical or prospective event or a specific weather context of interest) in traffic. In this light, the time periods along a given calendar with similar situational context (events of same magnitude or comparable weather) can be marked. As a result, multiple OD tensors sharing comparable context are produced. Differences between OD entries can be extracted using principles from contrast set mining [27] or visually highlighted [22]. Difference analysis can be done on traditional OD variables, such as passenger volume, as well as on variables capturing changes on the average time taken to complete a given OD trip or the adopted transportation modes. The gathered differences between context-sensitive and insensitive OD trips provide a simple yet effective way of supporting context-oriented mobility plans, such as decisions related with public transport strengthening needs in the presence of impactful events.

Context-aware tasks along the second scenario—OD tensor series—generally resort to the introduced principles for context-aware time series analysis. To capture the inherent spatial nature of OD data, particular attention should be placed to guarantee that the selected learning approaches are able to capture dependencies across series (OD entries). In this context, cross-series correlation factors can be explored in distance-based approaches using multivariate distances with cross-variable dependencies [2] and in neural processing approaches using spatiotemporal convolutions or graph representations [32].

#### Context-aware analysis of raw traffic data

Temporal association rules can be inferred from traffic events for the purpose of describing (forecasting) available (upcoming) traffic events [24]. The learning of these rules can be conditional to historical or prospective context in order to consider meaningful temporal dependencies between events for a given context. In addition, context-aware generative approaches placing Markov assumptions, such as hidden Markov models, can be also considered to model order dependencies between events for either describing available data or predicting the most probable event occurrences [14, 31]. For instance, considering events to be given by smart card validations, a generative model can be learned from a single passenger in order to abstract its daily transportation choices. The same probabilistic model can then be used to understand the most probable choices for a given day under a specific context. Context-aware analysis of raw card validations is also important to guide the estimation of entry or exit validations in carriers with non-mandatory entry or exit validations, as well as to generally impute missing data [43]. Illustrating, depending on the weather, a given user may change its transportation preferences, affecting the patterns of transportation usage. As such, the earlier introduced segmentation-based and correction-based principles can be further applied towards entry-exit estimators. The principles listed along Sect. 4.2.1 can be further extended to learn descriptors and predictors from traffic events. The inherent flexibility and suitability of deep learning approaches for event data make them also a good candidate for context-aware analysis by virtue of adding both static and temporal context variables [6, 49]. In this light, dedicated architectures, data mappings of event data to sparse time series, and graph-based views to simultaneously capture spatiotemporal dependencies are available in literature. In addition to deep learning approaches, context-aware distance-based approaches can be parameterized with distance functions able to assess the similarity between sets of events [12].

## Results

Considering the city of Lisbon as the case study, this section explores the impact produced by heterogeneous sources of situational context – planned events (Sect. 5.1), traffic interdictions (Sect. 5.2), weather records (Sect. 5.3), traffic generation/attraction poles (Sect. 5.4) and calendrical context (Sect. 5.5)—in different modes of transport, including subway (METRO), bus (CARRIS), public bike sharing (GIRA) and private road traffic.

### Planned events

**Fig. 6:**
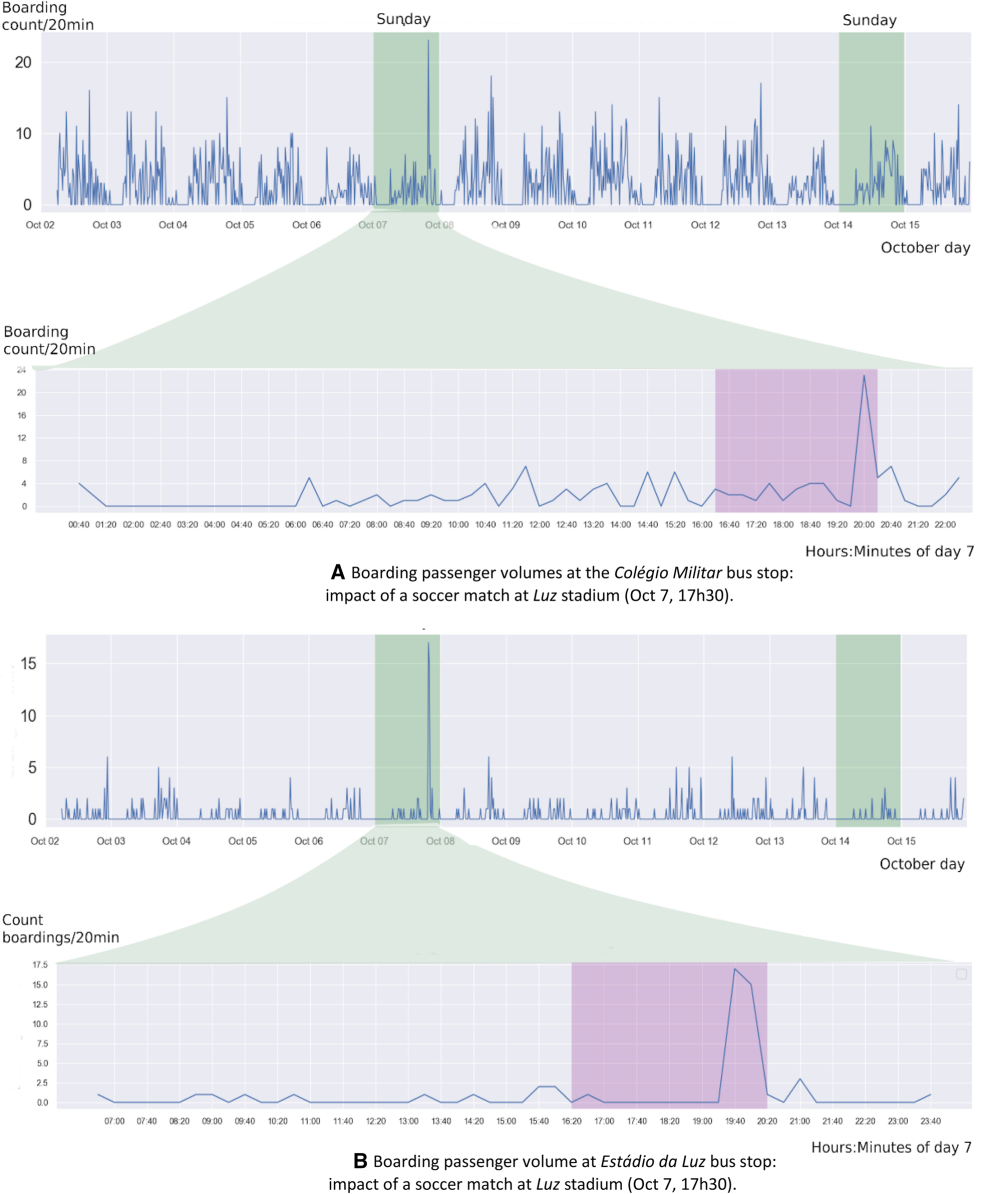
**a** Boarding passenger volumes at the *Colégio Militar* bus stop: impact of a soccer match at *Luz* stadium (Oct 7, 17h30). **b** Boarding passenger volume at *Estádio da Luz* bus stop: impact of a soccer match at *Luz* stadium (Oct 7, 17h30)

**Fig. 7:**
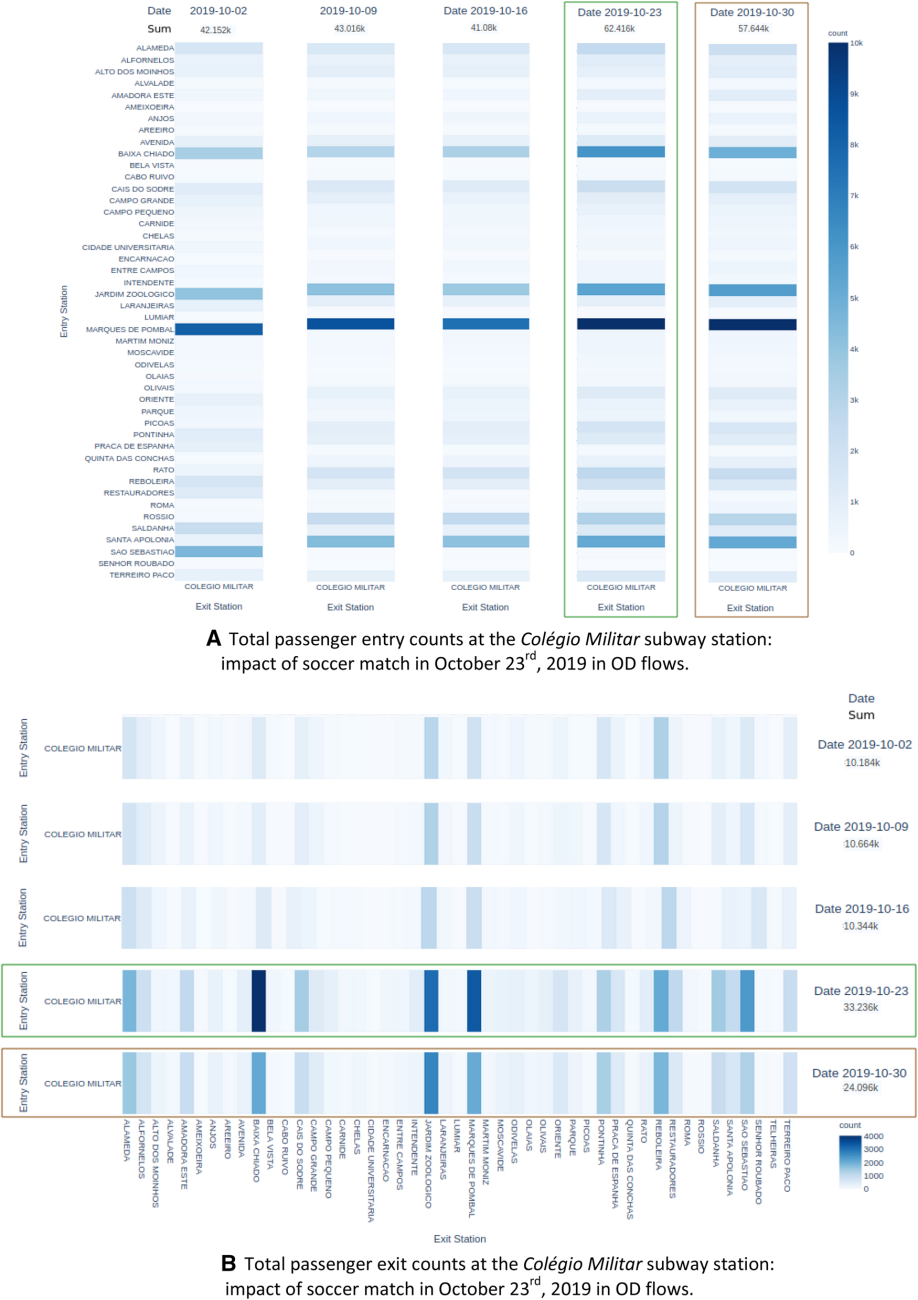
**a** Total passenger entry counts at the *Colégio Militar* subway station: impact of soccer match in October 23rd, 2019 in OD flows. **b** Total passenger exit counts at the *Colégio Militar* subway station: impact of soccer match in October 23rd, 2019 in OD flows

**Fig. 8:**
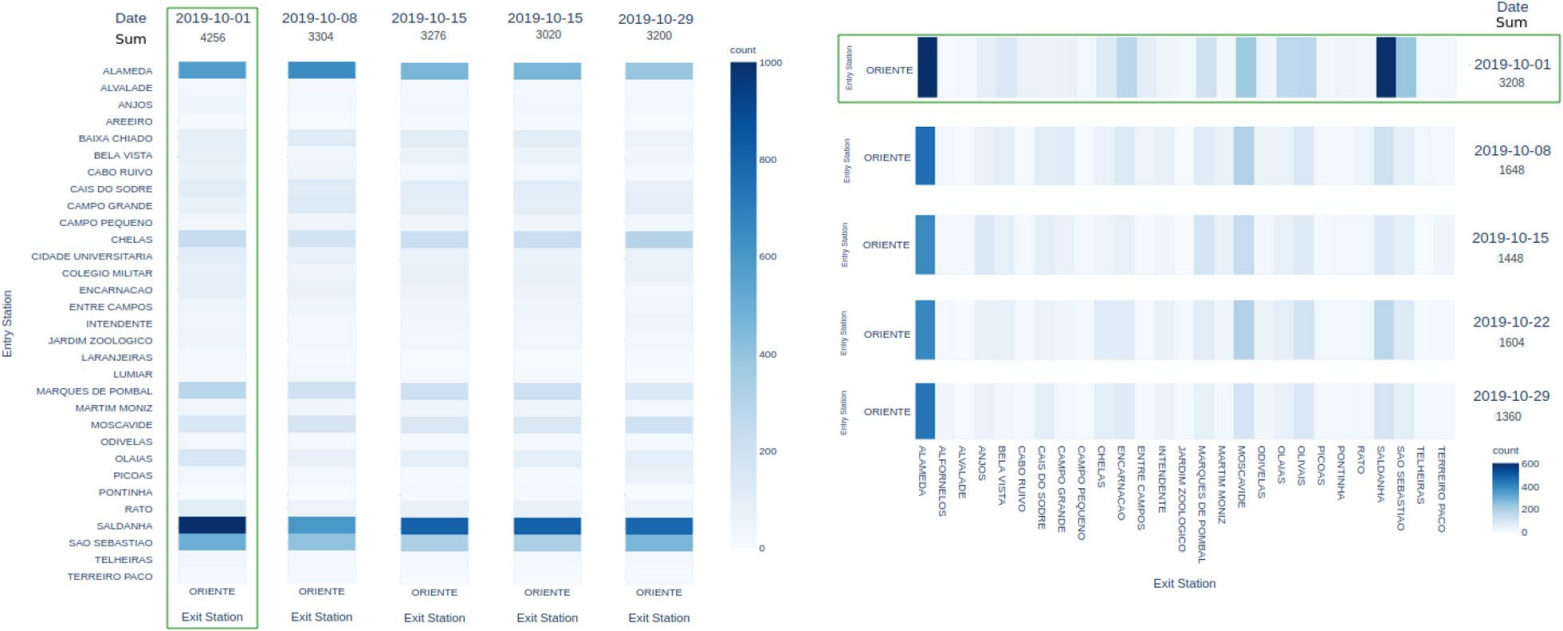
Total passenger exit and entry counts, respectively, at *Oriente* subway station: impact of concert in October 1st, 2019

Figures 6, 7 and 8 measure the impact yield by different public events—sport matches and concerts—in different transport modes—bus (CARRIS) and subway (METRO)—using different traffic data structures—time series and OD data.

Figure 6 depicts the impact generated by a soccer match in the volume of passenger boardings in two bus stops of *CARRIS* (the major bus carrier in Lisbon) near to the "*Luz*" stadium in the period between October, 2 and October, 15, 2018. The soccer match occurred in Sunday, October 7 at 17h30. The subsequent Sunday is highlighted to facilitate comparisons, providing strong evidence of the disruptive nature of the event in passenger demand. The sudden passenger peak could be easily mistaken as a statistical outlier in the absence of context data.

Figure 7 shows the impact of the alternative soccer matches when considering OD matrices dynamically inferred from paired entry-exit card validations at METRO (subway operator). The targeted soccer matches occurred along two consecutive Wednesdays–October, 23rd and 30th. Considering OD matrices inferred from identical weekdays in the presence and absence of the matches, the differences are clearly highlighted by the aggregate OD statistics or heatmap visualizations. In particular, Fig. 7a measures the entry volume of passengers between 4:00 pm and 8:00 pm in Wednesdays at *Colégio Militar* station, while Fig. 7b measures the exit volume of passengers at the same station between 9:00 pm and 11:00 pm. The difference on the volume generated between the two consecutive games is hypothesized to be associated with the popularity of matches—being the first match between *Benfica* and Lyon, *and* the second between *Benfica* and *Portimonense*—supporting the importance of considering the content of the event.

A similar analysis was carried out for the *Oriente* station, yet this time with the aim of measuring the impact on traffic demand generate by a large-scale concert. After detecting unusually high demand on this station on Wednesday, October 1 2019, the cultural records revealed the presence of a 2-h concert from Michael Bublé at the *Altice Arena* concert hall, in the immediacies of *Oriente* station. Figure 8 shows a considerable increase in passenger volume near the concert period. It is verified that there is, once more, a similarity between the patterns on the following Wednesdays after the event, while in the concert data an increase of 30 percentage points on exit (entry) passenger volume against the average was observed.

### Traffic interdictions

**Fig. 9:**
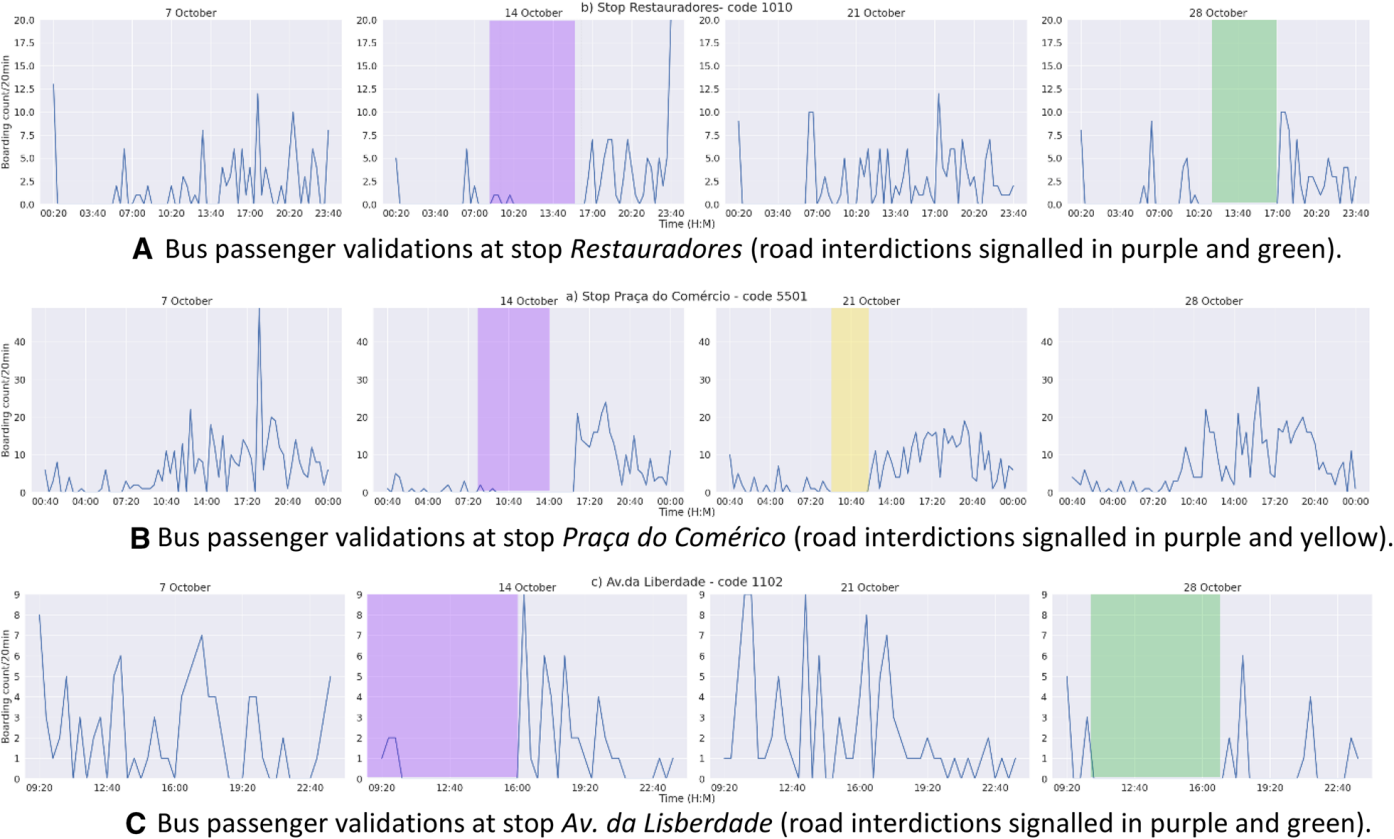
**a** Bus passenger validations at stop *Restauradores* (road interdictions signalled in purple and green). **b** Bus passenger validations at stop *Praça do Comérico* (road interdictions signalled in purple and yellow). **c** Bus passenger validations at stop *Av. da Liberdade* (road interdictions signalled in purple and green)

To highlight the relevance of context-based segmentation of data for traffic analytics, Fig. 9 quantifies the impact of three road closures and comparable interdictions in bus passengers’ validations. To identify the stops that are in the vicinity of the critical road segments, we created a tool that automatically detects the potentially affected bus stops and subway stations from planned closure segments, as well as their periods under the impact of the interdictions. Figure 4 captures interdictions along three consecutive Sundays due to the occurrence of special events in the city, each assigned with a colour in graphic visualizations. On October 14, between 8am and 14 pm, a marathon produced interdictions nearby the bus stops "*Restauradores*" and “*Av. da Liberdade*", with the finish line being placed near the bus stop "*Praça do Comércio*". This interdiction is represented in purple colour. On October 21 at 10 am, a bike event was acclaimed at "*Praça do Comércio*". This interdiction is represented with yellow color. On October 28 at 12am, an event inserted in the repository without content disclosure occurred at "*Restauradores*" and "*Av. da Liberdade*". The associated interdiction is represented in green. For this analysis, we consider passenger volume time series at three stops: "*Restauradores*" (Fig. 4a), "*Praça do Comércio*" (Fig. 4b), and "*Av. Liberdade*" (Fig. 4c). The figures show strong evidence in favour of traffic disruption, hence supporting the relevance of correctly annotating these periods to prevent demand deviations from affecting the subsequent learning of models of traffic data. On day 14, we observe zero boardings in the three locations during the marathon duration due to vehicle blockage, enclosed by peaks associated with the trips to the event and to return home. On day 21, boardings are further impacted at "*Praça de Comércio*" around 10am, and on day 28 at "*Restauradores*" and "*Av. da Liberdade*" after 12am.

**Fig. 10:**
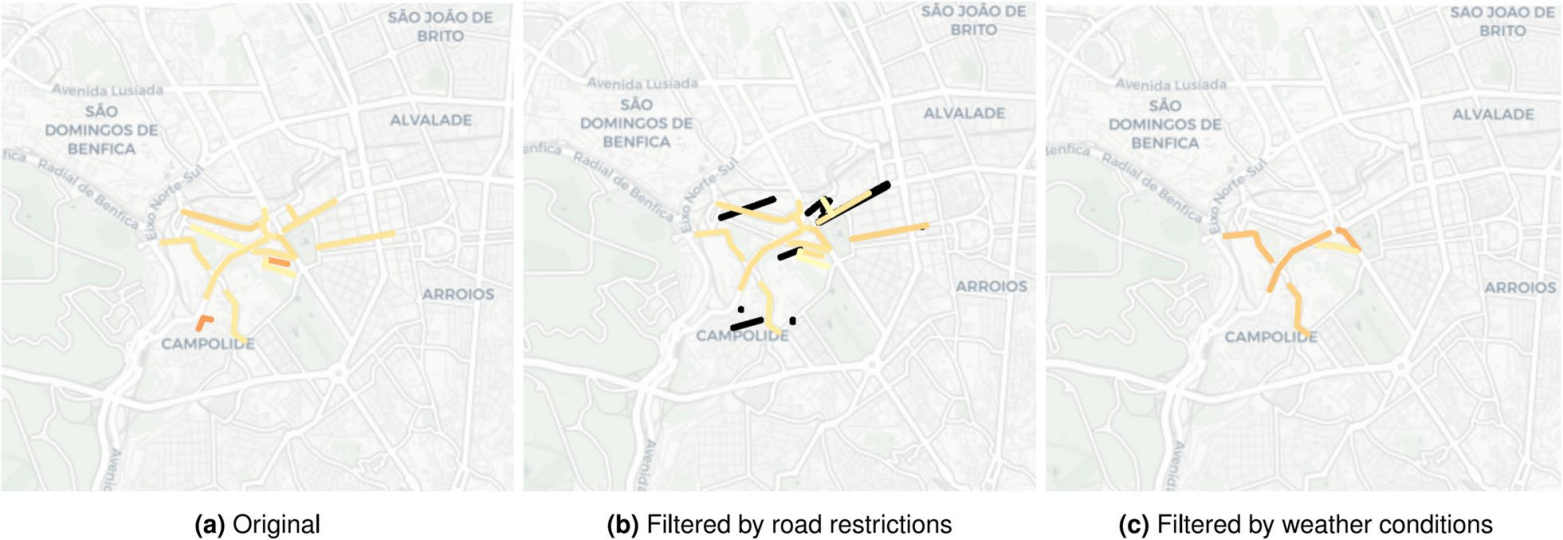
Map visualization of geolocalized speed data: **a** without situational context; **b** filtered by road restrictions (visually depicted in black); and **c** filtered by air humidity superior to 80%

Figure 10 further provides a view on how traffic congestions in the West side of the Lisbon city are affected under different locational contexts. Traffic congestions are inferred from individual road trajectories (geolocalized speed data) obtained from WAZE application during the second semester of 2019 [28].

### Urban planning: traffic generation poles

To assess the impact that traffic generation and attraction poles exert on the demand along the public transportation network, we provide the possibility to visualize maps of the passenger volume for the different carriers within the city of Lisbon together with a wide-diversity of poles with potential impact on traffic dynamics.

**Fig. 11:**
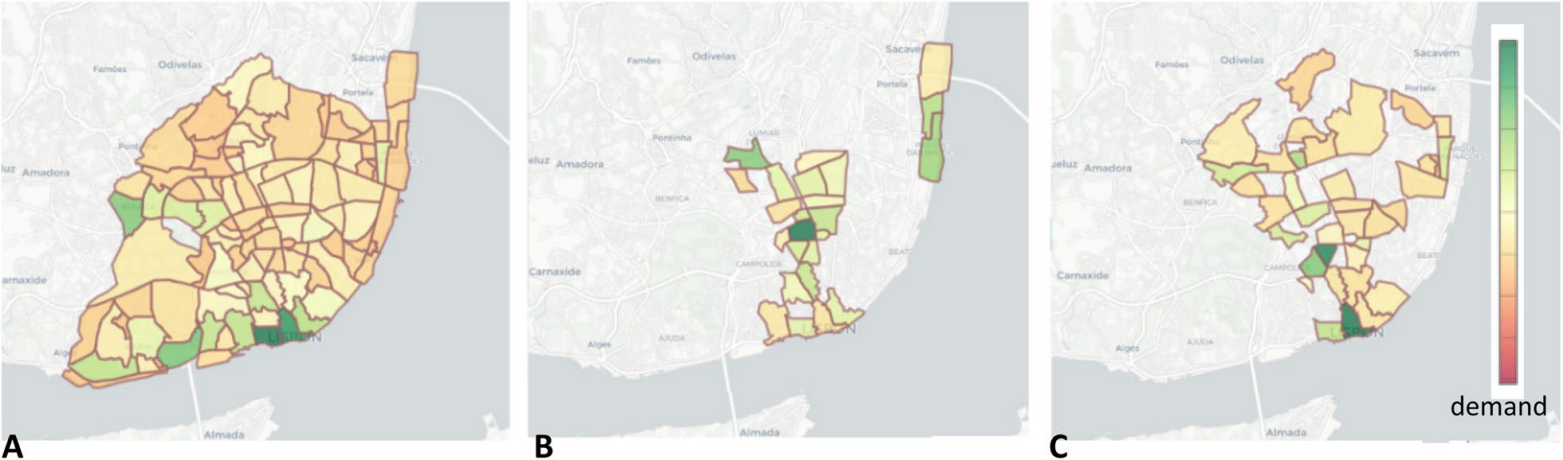
**a** Bus demand distribution; **b** Bike demand distribution; **c** Subway demand distribution

Figure 11 shows the geographical distribution of the passenger demand during 2018 for three distinct modes of transport: (1) bus (CARRIS) in Fig. 11a, (2) bicycle (GIRA) in Fig. 11b, and (3) subway (METRO) in Fig. 11c. As spatial criteria, we consider the TAZ (traffic analysis zoning) schema, providing geographical units used in transportation planning models.

**Fig. 12:**
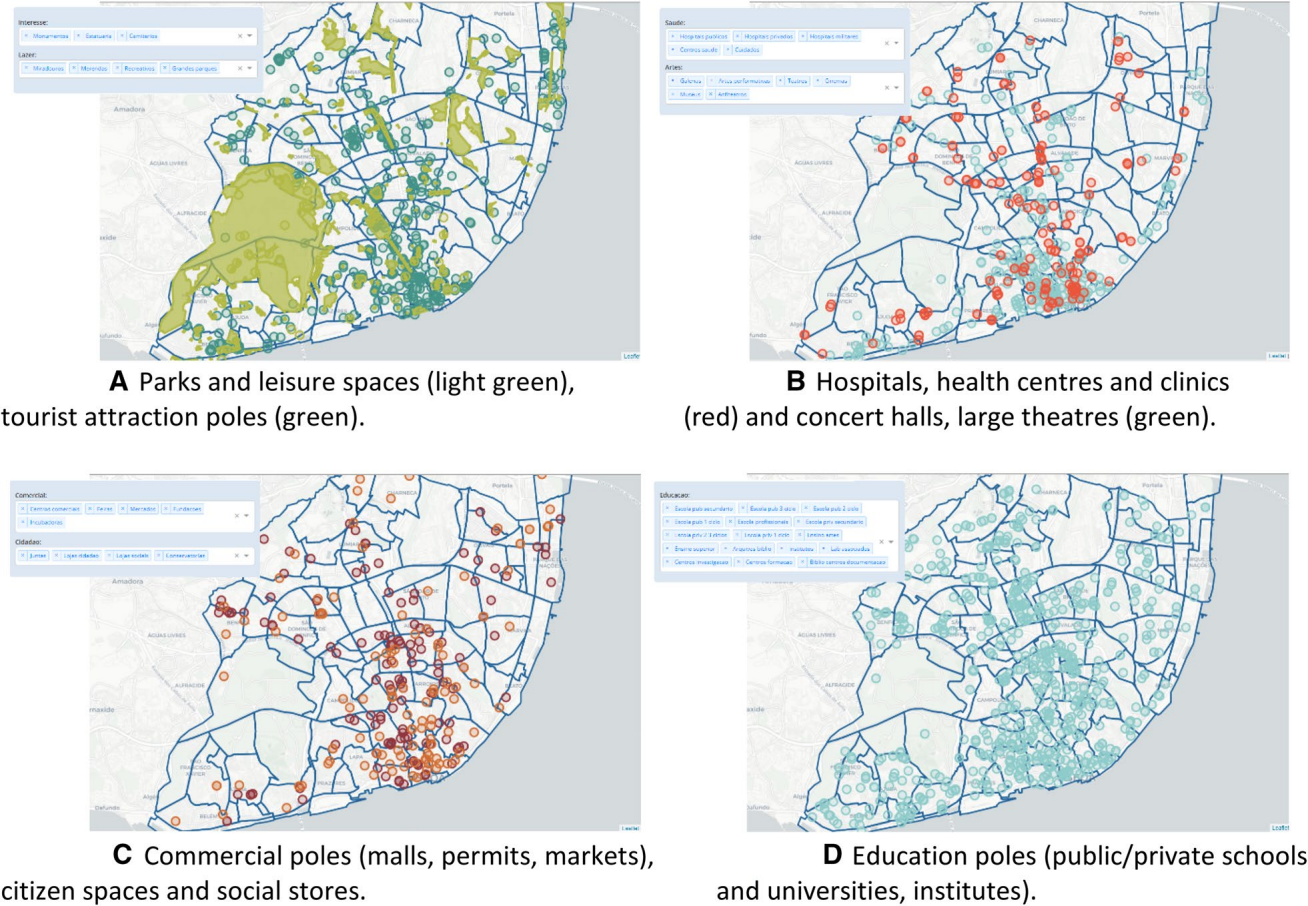
**a** Parks and leisure spaces (light green), **b** Hospitals, health centres and clinics tourist attraction poles (green). (red) and concert halls, large theatres (green). **c** Commercial poles (malls, permits, markets), **d** Education poles (public/private schools citizen spaces and social stores and universities, institutes)

Gathered views on transport demand and traffic congestion can be augmented with information pertaining to the available situational context, including city poles with capacity to attract generate traffic. Figure 12 provides a comprehensive listing of those poles including: tourist and leisure poles (Fig. 12a), health-related can cultural poles (Fig. 12b), commercial poles (Fig. 12c) and education and institutional poles (Fig. 12d). The presence of business, commercial and education poles is significantly correlated with the presented distribution of public transport demand in Fig. 5.

### Meteorological context

**Fig. 13:**
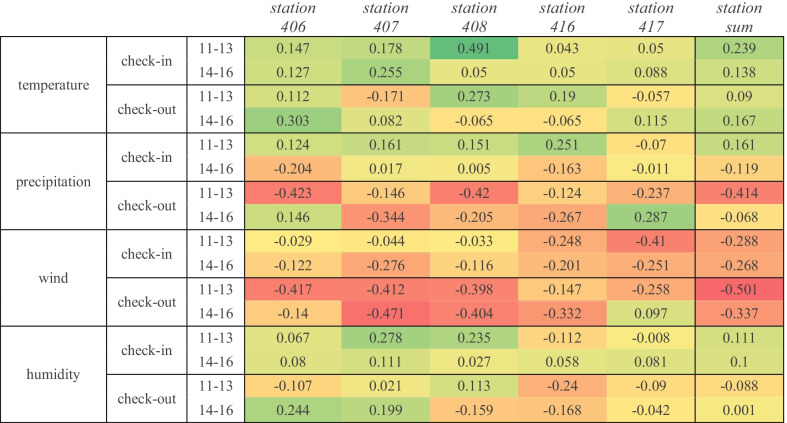
Pearson correlation between the weather data and the check-ins and check-outs at GIRA bike stations for intervals of 2 h in a day (11 h to 13 h and 14 h to 16 h) using data from 7/1/2019 to 28/2/2019

Figure 13 assesses the impact of weather on bicycle demand for the first time in the Lisbon city. This analysis provides a paradigmatic example of how correction factors can be approximated and subsequently considered to improve predictive models of bike sharing demand. To this end, data from the public bike sharing system in Lisbon (GIRA) and three weather stations were explored. The correlation using the simplistic Pearson coefficients after removing seasonal factors in the bike check-in and check-out demand is represented in Fig. 13. The GIRA stations chosen for the analysis were the stations with identifiers 406, 407, 408, 416 and 417, located in the Saldanha roundabouts and the area behind Instituto Superior Técnico. For the Pearson correlation analysis, we consider two off-peak periods—from 11 to 13 h and 14 h to 16 h—along working days. Periods with missing data were also removed. We can observe in Fig. 13 from the depicted Pearson correlations that bike demand is positively (yet softly) correlated with the temperature and negatively correlated with the wind intensity. No delineated correlations were found for the precipitation and humidity.

**Fig. 14:**
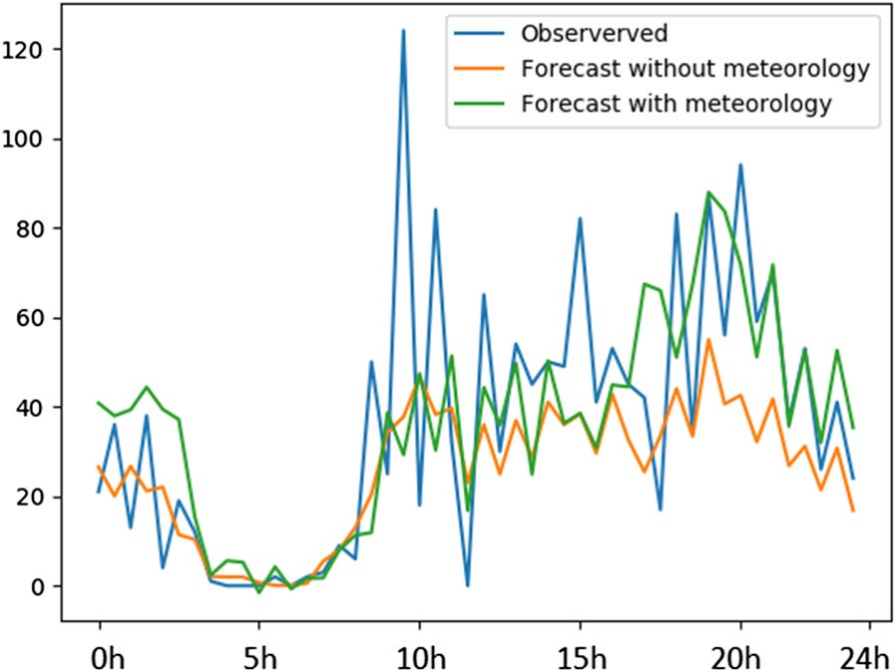
Context-aware LSTM forecasts for an aleatory testing data instance in the absence and presence of meteorological context: impact of historical and prospective weather in the forecasting of check-ins at GIRA network stations

Complementarily, Fig. 14 and Table 1 show the practical impact of considering weather context data to support forecasts of bike check-in demand at the GIRA network. To this, we consider LSTM forecasters in the absence of context and weather-enriched LSTM forecasters using the principles introduced in Sect. 4.2.1 to account for both historical and prospective weather records. These analyses show that the incorporation of meteorological context clearly guides forecasting for the selected time series. According to Fig. 8, the magnitude of errors against true observations along the horizon of prediction significantly decreases in the presence of weather records. These improvements are comprehensively assessed in Table 1.

**Table 1:** Comparison of context-unaware versus context-unaware state-of-the-art approaches of time series forecasting: root mean squared error (RMSE) after rolling cross-validation associated with the forecasting of check-ins at GIRA’s network (all stations) and single stations—details in [35]

Approach	RMSE	
	Network	Single station
KNN—DTW	23.52 ± 3.84	0.79 ± 0.14
KNN—Euclidean	24.06 ± 6.02	0.78 ± 0.14
Barycenter—DBA	27.87 ± 4.86	0.85 ± 0.15
Barycenter—Euclidean	24.81 ± 5.35	0.70 ± 0.21
Barycenter—DTW	26.87 ± 4.92	0.71 ± 0.22
Holts-Winters	47.42 ± 14.88	1.99 ± 0.73
LSTM	21.20 ± 3.40	0.72 ± 0.14
LSTM all meteo	20.94 ± 3.52	0.68 ± 0.14

### Calendrical context

**Fig. 15:**
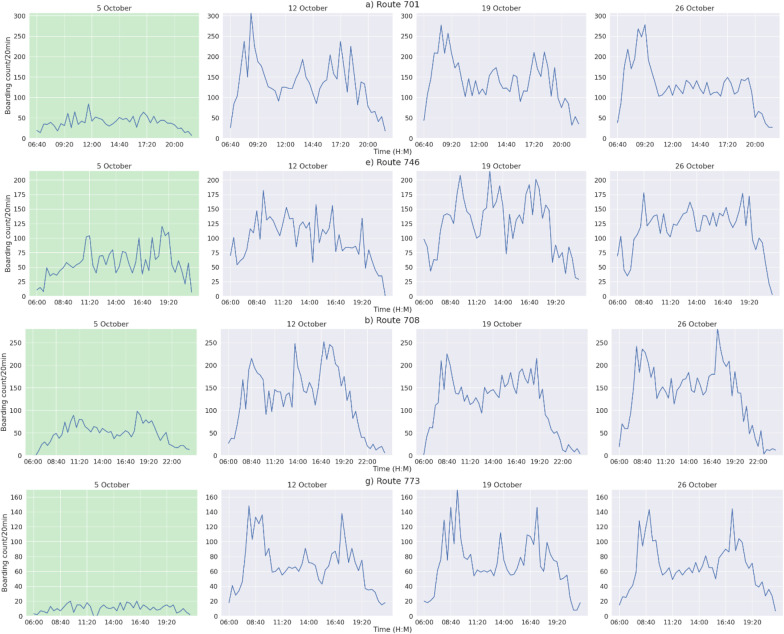
Effect of a national holiday (October 5) on passenger volume along four different bus routes

Classical approaches to traffic data analysis are generally sensitive to seasonal factors, including daily, weekly and yearly seasonalities, while machine learning approaches implicitly accommodate these factors in the learned associations from abundant batches of time series segments. Still, the arbitrary presence of festivities, local holidays or even academic calendars (on-academic and off-academic periods) are often disregarded, thus generally hampering traffic data analysis. Figure 15 provides a simple illustration of how a bank holiday can affect the average daily volume of passengers along different bus routes. Each route is subdivided into four time series, representing the same week day (Friday). A national holiday (Friday, October 5) is highlighted in green. The drop in passenger demand is extremely marked and should be carefully accounted not to hamper the learning of the target descriptive and predictive models.

**Fig. 16:**
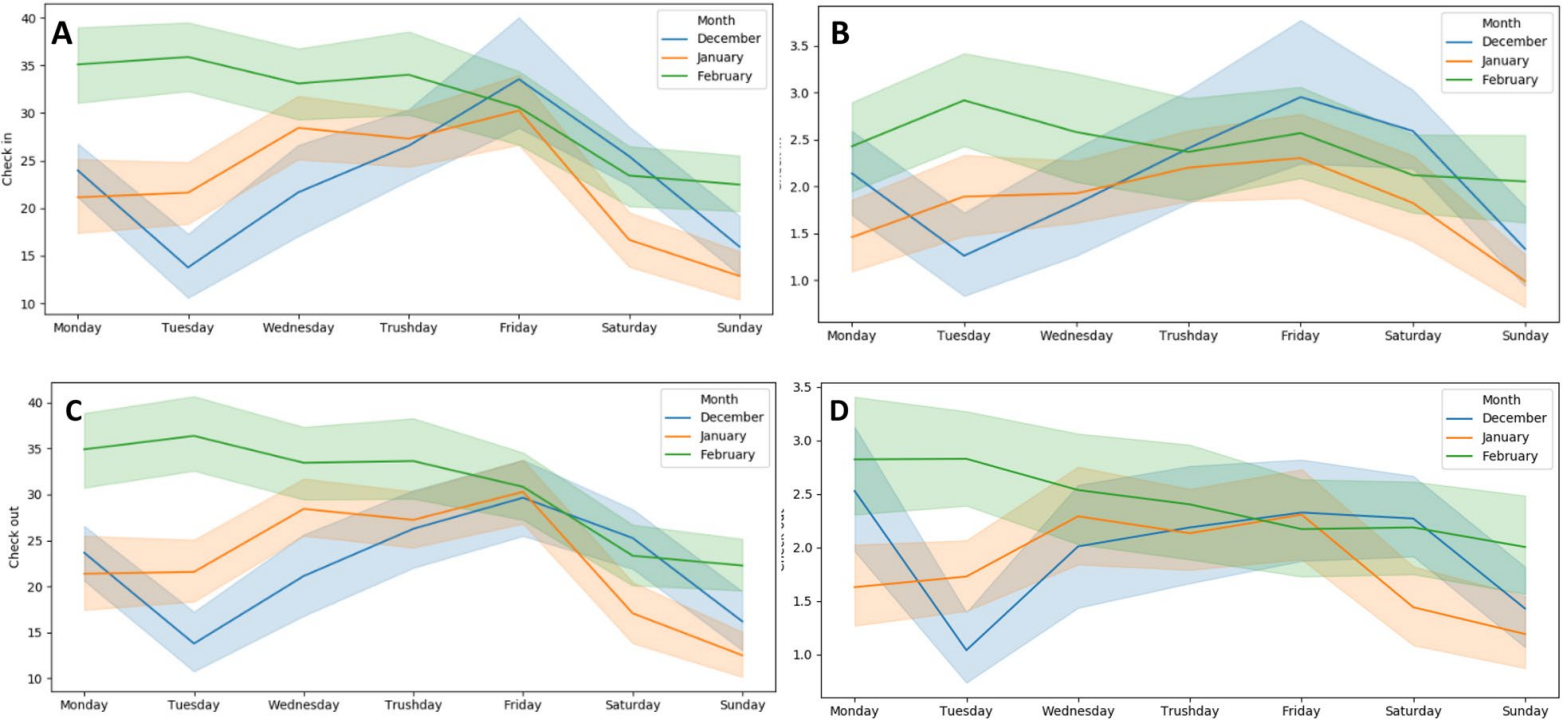
Volume of check-ins and check-outs for all GIRA bike stations (**a**, **b**) and the IST cluster of three stations (**c**, **d**): average number per weekday (and associated bounds) along three consecutive months

Complementarily, Fig. 16 provides the average daily volume of check-ins and check-outs at the stations of the Lisbon’s public bike sharing network (GIRA). In particular, we show the average number of check-ins (Fig. 16a, c) and check-outs (Fig. 10b, d) for each weekday along four months (December 2018 to March 2019 after correcting for holiday and festivity periods). The analysis of demand is shown for all the stations in Lisbon (Fig. 16a, b) and for a cluster of stations nearby *Instituto Superior Técnico* (Fig. 16c, d). This analysis clearly shows that traffic dynamics clearly vary among weekdays (with peaks generally observed on Mondays and Fridays), highlighting the importance of accounting their differences in traffic data analysis, whether through calendric-guided segmentation, correction factors, or masking principles [35].

## Conclusions and discussion

Moved by the undoubted observation that accessible contextual factors strongly shape public transport demand, this work provides a structured view on how to incorporate heterogeneous sources of situational context into traffic data analysis for urban mobility planning. To this end, we surveyed and discussed important methodological principles towards the integrative analysis of traffic data and its situational context in accordance with:

i) The selected modes and sources of traffic data and their representation, whether through georeferenced time series, origin–destination (OD) tensors or raw traffic events;
ii) The type of context, whether historical or prospective, and whether situational, meteorological or/and calendrical;
iii) The targeted task, whether descriptive or predictive; and
iv) The pursued principles for the context-augmented analysis of traffic data, whether given by *context-driven traffic corrections*, *context-driven traffic data segmentation* or *context-sensitive learning approaches*.

Using the city of Lisbon as a case study, we further motivate the role of well-established initiatives towards the collection of a wide-diversity of sources of relevant urban context into (semi-)structured repositories, allowing the dynamic acquisition and consolidation of both historical and prospective context information. In this light, multivariate, temporal and spatial urban content is standardly assigned to context records, allowing the real-time augmentation of traffic data using simplistic online routines to fetch new or updated context records. For instance, a comprehensive list on the requested occupancies of city halls, theaters, congress rooms, exposition centers, stadiums, and other large facilities is maintained in real-time. Similarly, historical and prospective geospatial information on road interdictions and other complementary events with potential impact on urban mobility are maintained by the city Council. In this work, the consolidation of context and traffic records is established via a multi-dimensional model in accordance with their spatial and temporal footprint.

Once context data is consolidated, context-guided modelling of traffic dynamics becomes possible. In particular, the gathered results further motivate the relevance of context-sensitive correction, segmentation and learning principles for traffic data analysis. As extreme weather conditions affect transport modal preferences, we saw that weather-demand correlations can be assessed and used to discount predictive models of cycling demand with positive impact in the city of Lisbon. The analysis of congestions in road traffic data is currently undertaken by segmenting traffic in multiple comparable periods in accordance with calendrical constraints and additional available situational context. We further discussed how neural networks can be extended to incorporate both historical and prospective sources of context using masking principles for predictive tasks. To this end, prospective context, such as weather forecasts or planned events, can act as a regularizer of predictions and has shown to improve the forecast of station congestions at the Lisbon's public bike sharing system. Public transport reinforcement decisions for large-scale events, such as planned festivals and soccer matches, can further benefit from context-comparable origin–destination tensor data. CARRIS, the major bus operator in Lisbon, currently relies on dynamic multimodal origin–destination tensor data to assess demand deviations caused by public events. Concluding, the gathered results stress the importance of incorporating historical and prospective context data for a guided description and prediction of urban mobility dynamics, irrespective of the underlying data representation.

## Data Availability

The *context data sources* used in this study are available at Lisboa Aberta (https://dados.gov.pt/) and IPMA (Portuguese weather agency). The *traffic data* collected for the different transport modes (bus, subway, bike sharing system, road traffic) were accessed under NDA agreements with CML, CARRIS and METRO.

## Footnotes

1 ILU (Integrative Learning from Urban Data): http://www.iluproj.com/

2 https://www.gira-bicicletasdelisboa.pt
